# Enhancement of Optical Coherence Tomography Images Using Adversarial Neural Networks: Impacts on Ophthalmic Practice

**DOI:** 10.7759/cureus.93423

**Published:** 2025-09-28

**Authors:** Fernando Henrique F Teixeira, Valberto M Nunes, Brena Fernanda S Carvalho, Francisco Vinícius M Souza, José Leandro N Silva, Rafael Scherer, Fernando K Malerbi, Luis Nakayama, Alexandre Antonio M Rosa, Caio Vinicius S Regatieri

**Affiliations:** 1 Ophthalmology, Universidade Federal do Rio de Janeiro, Rio de Janeiro, BRA; 2 Ophthalmology, Universidade Federal de São Paulo (UNIFESP), São Paulo, BRA; 3 Ophthalmology, Instituto de Ciências da Saúde, Universidade Federal do Pará, Belém, BRA; 4 Ophthalmology, Bascom Palmer Eye Institute, University of Miami, Coral Gables, USA

**Keywords:** ai, artificial intelligence in healthcare, generative adversarial network (gan), medical retina, oct (optical coherence tomography), optical coherence tomography (oct)

## Abstract

Purpose: The integration of artificial intelligence (AI) into ophthalmology has rapidly advanced, particularly in the analysis of optical coherence tomography (OCT) images. OCT is a non-invasive imaging modality widely used to visualize retinal layers for diagnostic and therapeutic purposes. Recent advancements suggest that AI can enhance image resolution, interpret data more effectively, and improve clinical decision-making. This study's purpose is to evaluate the application of an AI-driven filter to enhance OCT images, focusing on its impact on image clarity and diagnostic accuracy.

Methods: We utilized generative adversarial networks (GANs), specifically the real-enhanced super-resolution GAN (Real-ESRGAN), to enhance the resolution and clarity of OCT images. The study included eight anonymized OCT images from the Ophthalmology Department at the Universidade Federal de São Paulo (UNIFESP), featuring various retinal pathologies. A total of 147 ophthalmologists (96 retinal specialists and 51 non-specialists) assessed the images before and after AI enhancement. Descriptive statistics, contingency tables, and chi-squared tests were employed to analyze the data, using the Jamovi software (jamovi (Version 2.3) [Computer Software]. Retrieved from https://www.jamovi.org) and the R software (R Foundation for Statistical Computing, Vienna, Austria) with a significance level set at 5%.

Results: The majority of ophthalmologists (69.4%) found that AI-enhanced images improved the clarity of retinal layers, with external retinal structures being particularly well-evaluated. However, opinions varied regarding the clinical utility of AI-enhanced images in diagnostic and prognostic contexts. While 61.9% of participants agreed that AI facilitated biomarker identification, 60.5% did not believe it provided additional relevant information for case management. Notably, AI-enhanced images were considered to significantly highlight biomarkers, with 78.9% of ophthalmologists acknowledging this benefit.

Conclusions: AI-enhanced OCT images, particularly those processed with GANs, can significantly improve the clarity and detail of retinal images, which may aid in more precise diagnostics. Despite the positive feedback, the variability in perceived clinical utility suggests that AI integration should be approached cautiously. Training and experience in using AI tools are crucial, and further research with larger samples and diverse clinical settings is needed to better understand AI's impact on clinical practice and its cost-benefit ratio. Future studies should explore the comparative efficacy of different AI algorithms and their potential to enhance diagnostic accuracy and patient outcomes.

## Introduction

Ophthalmology is at the forefront of medical specialties adopting artificial intelligence (AI) in its various forms. This is primarily due to its imaging-centric nature. In this specialty, the evaluation of imaging exams and data interpretation play a fundamental role in diagnosing, prognostic assessing, and treating diseases [1].

Optical coherence tomography (OCT) is a non-invasive examination modality that provides high-speed tomographic images of different human tissues. In ophthalmology, this technology is routinely utilized to visualize the various layers of the retina for diagnostic and therapeutic purposes. Recent studies have shown that OCT scanning has benefited from AI for improving image resolution, data interpretation, and the accuracy of clinical decisions [2]. AI models can detect and delineate, for example, retinal fluid in conditions such as central retinal vein occlusion, age-related macular degeneration (AMD), and diabetic macular edema [2]. Furthermore, AI can detect exudative AMD and predict its progression [3].

Thus, the integration of innovative technologies, such as AI, into healthcare can potentially bring significant advances in disease diagnosis and treatment. By employing AI to assist in the interpretation of imaging examination results, technologies can analyze vast amounts of data, identify subtle patterns, and provide more accurate and timely diagnoses which are expected to emerge [2,3]. This advancement will directly impact the quality of healthcare offered to patients, enabling earlier, faster, and more accurate diagnoses of ocular diseases, ultimately leading to more appropriate treatments and better outcomes for patients.

In this study, we propose the use of a filter employing AI technology to enhance image details, remove noise, and enhance certain retinal structures, particularly the outer layers. This study aimed to evaluate the effectiveness of a generative adversarial network (GAN) in enhancing the resolution and clarity of OCT images, comparing the perception of experts and non-experts in this field.

## Materials and methods

Methods

GANs represent an advanced class of AI models that have revolutionized the ability to generate high-quality synthetic data. Structurally, GAN comprises two different neural networks, trained in an adversarial fashion: (1) the generator and (2) the discriminator. On the one hand, the generator is a neural network responsible for generating new synthetic data. For images, for example, the generator creates images that resemble the original dataset but are not directly taken from it. Initially, the generator produces random data; however, through training, it generates data that becomes increasingly similar to real data. On the other hand, the discriminator is a network that assesses whether a given data sample is real (originating from the real dataset) or synthetic (produced by the generator). It is trained with examples of both types of data to improve its ability to distinguish between them during training.

During GAN training, the generator and discriminator are trained in parallel and adversarial pathways: the generator aims to create data that deceives the discriminator by being as real as possible, whereas the discriminator tries to distinguish between real and generated data.

This competitive process allows for improvement in the competency of both the generator and discriminator until the generator can generate synthetic data that is indistinguishable from real data, at least from the discriminator's perspective [4].

**Figure 1:**
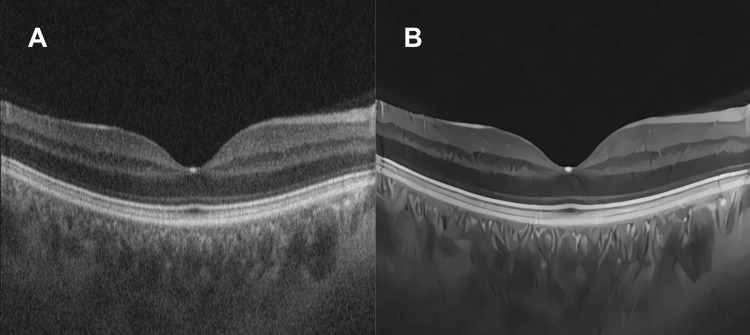
(A) Normal macula OCT. (B) OCT of the macula within the normal range post-treatment with the AI algorithm. OCT: optical coherence tomography; AI: artificial intelligence

In this study, a GAN specifically trained using anime drawings was used to enhance image resolution [5] and was later successfully adapted to enhance OCT images of the retina (Figure 1). By applying super-resolution and noise removal techniques, such as those developed by real-enhanced super-resolution GAN (Real-ESRGAN), the GAN can process OCT images and significantly increase their visual quality. This model was originally trained to address complex real-world image degradations using a residual-in-residual dense network architecture.

Anime images are characterized by having a more pronounced contrast compared with real photographs, mainly due to sharp lines and the extensive use of shadows and highlights. These features have been harnessed and applied to facilitate the visualization of the various layers of the retinal tissue in sharper and more precise detail, allowing ophthalmologists (retinologists and non-retinologists) and AI to identify areas of interest and potential abnormalities.

Materials

**Figure 2:**
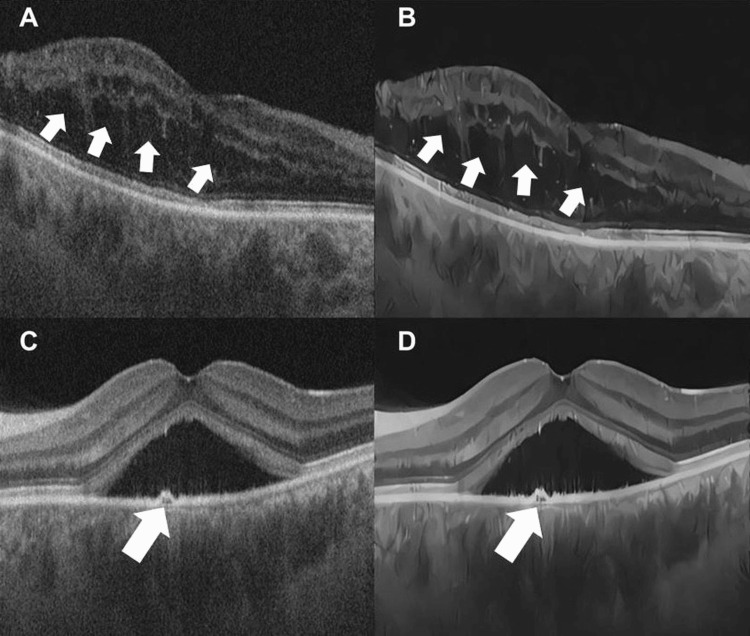
(A) OCT of the macula showing macular edema (white arrows). (B) OCT of the macula showing macular edema after treatment with the AI algorithm (white arrows). (C) OCT of the macula in central serous chorioretinopathy with a small serous detachment of the retinal pigment epithelium (white arrow). (D) OCT of the macula in central serous chorioretinopathy after treatment with the AI algorithm (white arrow). OCT: optical coherence tomography; AI: artificial intelligence

**Figure 3:**
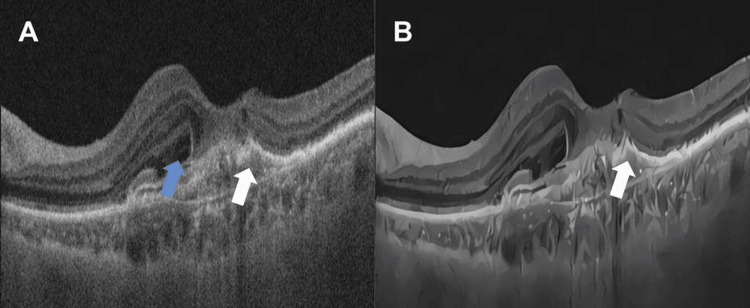
(A) OCT of the macula showing a type 1 subretinal neovascular membrane with the presence of a fibrovascular detachment of the retinal pigment epithelium (white arrow) and subretinal fluid (blue arrow). (B) OCT of the macula showing a subretinal neovascular membrane after treatment with the AI algorithm (white arrow). OCT: optical coherence tomography; AI: artificial intelligence

Eight de-identified retinal OCT images were collected from the image bank of the Ophthalmology Department at the Universidade Federal de São Paulo (UNIFESP), São Paulo, Brazil. The images pertain to the examinations of patients with various ocular pathologies, such as central serous chorioretinopathy and neovascular membrane. These specific images were selected because these diseases predominantly affect the outer retina, especially structures that can be identified and made clearer by AI technology (Figure 2 and Figure 3). Among the imaging biomarkers evaluated, notable examples include the integrity of the ellipsoid zone, the presence or absence of intraretinal cysts, pigment epithelial detachments, and subretinal hyperreflective material (SHRM).

The inclusion criteria for image selection were established to ensure sample homogeneity and data quality. Patients aged 18-75 years whose OCT images showed adequate transparency of the ocular media and refractive error (spherical equivalent) between ±3.50 diopters were included to minimize distortions in result interpretation. Conversely, patients who had undergone posterior vitrectomy at any time were excluded, as this condition significantly alters retinal anatomy and compromises the quality of the images obtained, impairing the validity of the data analyzed. These images were processed using GAN technology, and the results obtained before and after applying the algorithm were presented to 147 ophthalmologists.

A questionnaire was designed consisting of seven questions addressing aspects such as the clarity of the analysis of the image layers, assessment of specific structures that could be better visualized following AI use, and relevance of the additional information provided by AI treatment for the management of the clinical case. Furthermore, the questionnaire explored the facilitation of diagnosis and management offered by the AI filter, its efficacy in identifying biomarkers, and its contribution to a more accurate prognostic assessment. Finally, the perception of biomarker enhancement in imaging after AI application was investigated to understand how this technology impacts clinical practice.

Statistical analysis

A comprehensive statistical analysis was performed to investigate the benefit of using AI via GANs to enhance OCT-captured retinal images. Data were collected and analyzed using both descriptive and inferential statistical approaches to understand the associations and impacts of the variables studied.

Initially, data was collected from 147 ophthalmologists, with 96 (65.3%) being retinal specialists and 51 (34.7%) non-specialists. Participants were categorized based on several variables, including years of professional experience (classified as "0-10", "11-20", "21-30", and ">31" years) and perceptions on the use of AI in different aspects of image analysis, such as clarity of retinal layers, identification of biomarkers, facilitation of diagnosis, biomarker enhancement, facilitation of prognostic assessment, perception of relevant details, and better identification of ocular structures (choroid, vitreoretinal interface, and outer and inner retina).

Descriptive analysis was conducted to summarize and understand the distribution of the collected variables. Absolute and relative frequencies were calculated for each category of variables, providing an overview of the demographic and professional characteristics of the participants, as well as their perceptions regarding the use of AI in ophthalmology. This step allowed the identification of initial patterns and trends in data, establishing a foundation for further analysis.

To explore possible associations between categorical variables, contingency tables were constructed by cross-referencing variables such as retinal specialization with perceptions of AI efficacy in different clinical contexts. Chi-squared (χ²) tests were applied to assess the statistical significance of these associations. A p-value of <0.05 was considered statistically significant, indicating a non-random association between the examined variables.

To better understand the factors influencing the perception of retinal layer clarity provided by AI, a binomial logistic regression analysis was conducted. In this analysis, the dependent variable was "Perceived clarity of layers through AI" (classified as "Yes" or "No"), and the independent variables were retinal specialist, identification of biomarkers, facilitation of prognostic assessment, biomarker enhancement, perception of relevant details, and facilitation of diagnosis.

The variables for the final model were selected using the stepwise method based on p-values, progressively excluding variables from the model according to their statistical significance. This method ensures that only the most relevant predictors are retained, optimizing the simplicity and efficiency of the model.

Measures of fit, such as the Akaike information criterion and McFadden's R², were calculated to assess the quality of the model and its explanatory power. Estimated coefficients, standard errors, z-statistics, p-values, odds ratios (ORs), and 95% confidence intervals (CIs) were reported for each independent variable, allowing a detailed interpretation of the influence of each predictor on the dependent variable.

All statistical analyses were performed using the Jamovi software (jamovi (Version 2.3) [Computer Software]. Retrieved from https://www.jamovi.org), which provided a robust and flexible platform for advanced analysis, ensuring the accuracy and reliability of the results obtained.

## Results

Participants

In total, 147 ophthalmologists participated in the study by answering the questionnaire, of whom 96 were retinologists and 51 non-retinologists. The mean length of professional practice was approximately 12 years (standard deviation = 9.54).

**Figure 4:**
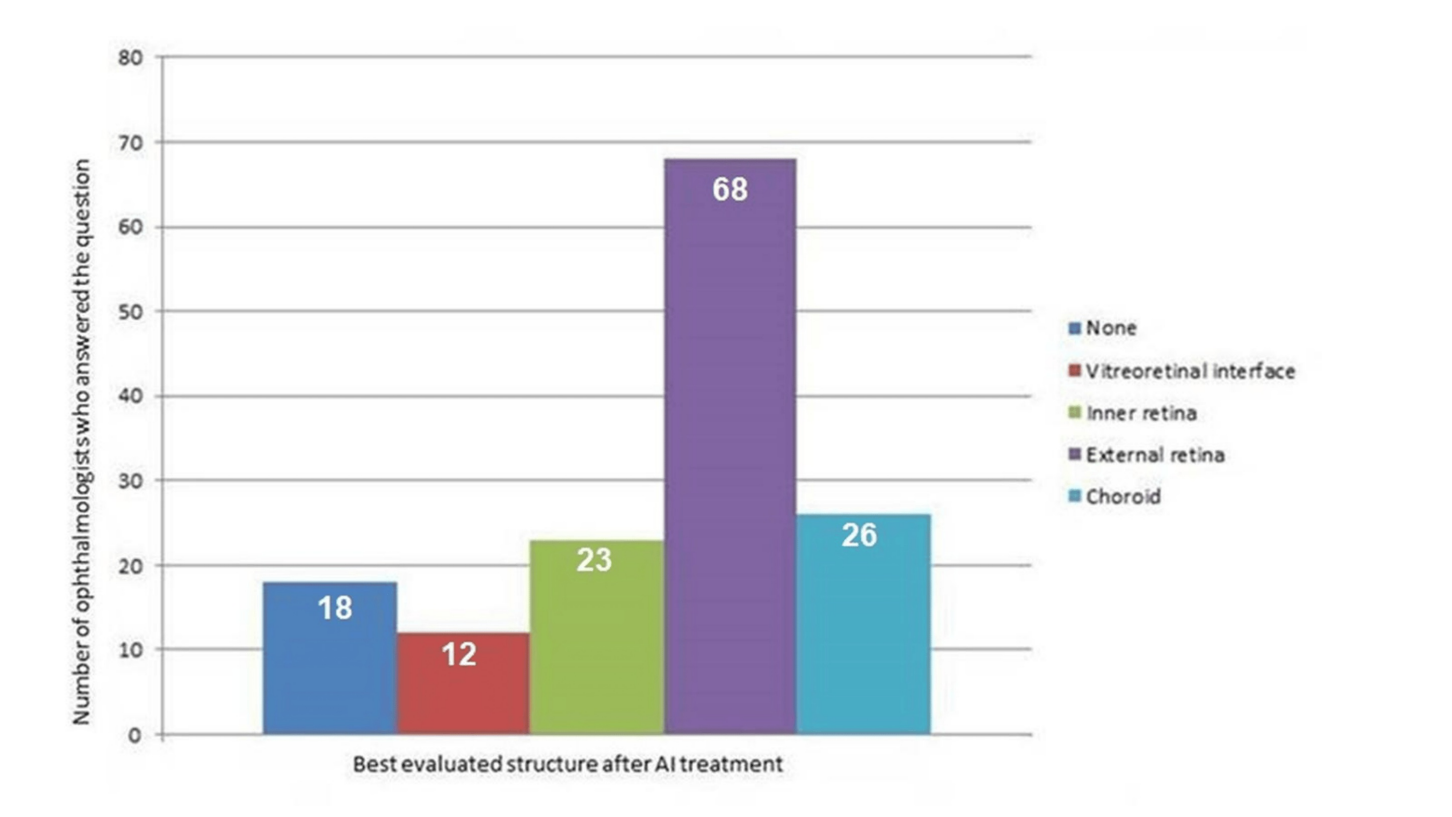
Best evaluated structure following AI treatment AI: artificial intelligence

**Figure 5:**
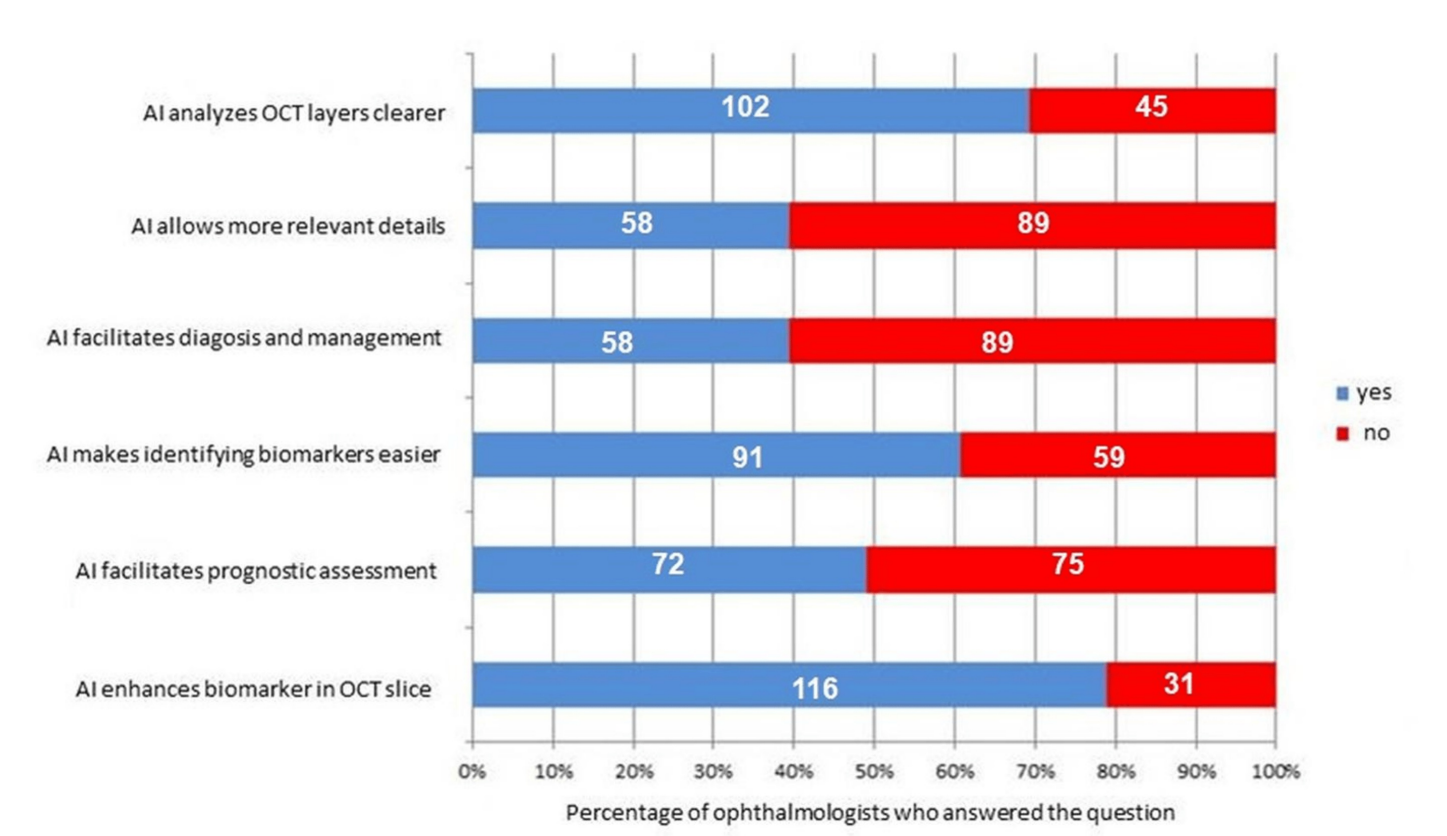
Answers to six different aspects involving the enhancement of OCT images following AI treatment OCT: optical coherence tomography; AI: artificial intelligence

Figure 4 and Figure 5 below present graphs displaying the results of the evaluations regarding the images processed with the algorithm.

Interpretation of results

The descriptive analysis revealed that most participants recognized the improvements associated with the use of the AI algorithm in image evaluation. Specifically, 69.4% of professionals stated that AI contributes to the clarity of retinal layers (Figure 4). The χ² association test between "Retinal Specialist" and "AI Layer Clarity" showed a p-value of 0.001, indicating a statistically significant association between these variables. This suggests that retinal specialists have different perceptions of the effectiveness of AI in improving retinal layer clarity compared with non-specialists. Specifically, a higher proportion of non-experts recognize the positive contribution of AI in this regard.

Logistic regression was performed to identify which factors are significantly associated with the perception that AI improves retinal layer clarity. The main results and interpretations are detailed below.

Coefficients and Significance of the Independent Variables

Being a retinal specialist (estimate: -1.690; OR: 0.185; 95% CI: 0.0595-0.573; p = 0.003) was associated with lower odds of realizing that the AI algorithm improves retinal layer clarity. The ORs show that retinal specialists are approximately 81.5% less likely to agree that AI improves the clarity of the retinal layers compared with non-experts. This result may suggest that specialists rely more on their clinical expertise or may be more critical of the improvements provided by AI.

For biomarker identification (estimate: 1.767; OR: 5.855; 95% CI: 2.2213-15.431; p < 0.001), professionals who believe that AI facilitates the identification of biomarkers are 5.85 times more likely to believe that AI improves the clarity of the retinal layers.

Regarding the facilitation of prognostic assessment (estimate: 1.075; OR: 2.931; 95% CI: 10313-8.328; p = 0.044), professionals who believe that AI facilitates prognosis are around 2.93 times more likely to perceive improvements in retinal layer clarity with the use of AI. This result highlights the interrelationship between the perception of AI as a comprehensive tool that improves both diagnosis and prognosis in ophthalmology.

For biomarker enhancement (estimate: 1.507; OR: 4.513; 95% CI: 14,157-14,387; p = 0.011), the belief that enhanced biomarkers were associated with a 4.51-fold increase in the odds of perceiving improvements in retinal layer clarity suggests that recognizing AI's ability to enhance specific aspects of retinal images contributes to an overall positive perception of its effectiveness in improving the visual quality of retinal layers.

## Discussion

The results obtained from the 147 participating ophthalmologists indicate that, although AI may potentially improve image clarity and detail, the perception of the influence of technology on clinical practice varies substantially among professionals. The predominant perception that AI can improve the visualization of the outer retinal layers, corroborated by most participants (Figure 4), suggests that AI-based algorithms and filters have the potential to contribute to a more accurate analysis of ocular structures [5].

The results of the logistic regression indicate that positive perceptions of AI's ability to identify and enhance biomarkers and facilitate prognosis are strongly associated with the belief that AI improves the clarity of the retinal layers. We observed that being a retinal specialist is associated with a lower probability of recognizing these improvements, which may reflect differences in clinical experience, expectations, or familiarity with AI technologies between specialists and non-specialists. However, the analysis of the length of experience as a retinal specialist was not statistically relevant to the perceived benefit of the AI algorithm.

These results suggest that the acceptance and perceived benefit of AI in ophthalmology may vary depending on the specialization and previous experience of professionals. Training and continuing education programs can be developed to increase retinal specialists' familiarity with and confidence in AI tools, highlighting their benefits in clinical practice.

Furthermore, the recognition that AI contributes significantly to the identification and enhancement of biomarkers (Figure 5) may encourage the greater integration of these technologies into clinical workflows, potentially improving diagnostic accuracy and the management of ocular diseases.

This study showed the promising impact of applying GANs on enhancing the quality of OCT images in ophthalmology. GANs are applied in several areas, such as high-resolution imaging, speech synthesis, and text generation, among others, and are particularly useful in scenarios in which generating realistic, high-quality data is challenging using traditional methods. The Real-ESRGAN model was used due to its robustness against typical imaging artifacts [6], such as noise and blurring, features also present in OCT images.

In ophthalmology, the application of GANs has transformative potential. Especially in the enhancement of medical images, such as those obtained by OCT, GANs improve the resolution and clarity of captured images. By training a GAN with a large dataset of high-quality images, the model can synthesize OCT images that are more visually accurate and useful for clinical diagnoses [1].

For instance, a GAN, such as Real-ESRGAN [6], can be used to promote super-resolution in OCT images to reveal microscopic details that would otherwise be imperceptible in low-resolution images. This improves ophthalmologists' ability to identify ocular pathologies early on as well as facilitates image interpretation and analysis, resulting in more accurate and effective diagnoses. Thus, GANs elevate the quality of medical imaging and promote significant advances in clinical practice, contributing to more advanced and personalized ophthalmologic care. These results reaffirm the usefulness of AI in improving and interpreting medical imaging exams, consistent with previous studies, even outside the field of ophthalmology. In radiology, for example, Hoppe et al. evaluated the implementation of AI for detecting fractures in emergency radiographs. The results showed that AI reduced missed findings and increased reader safety, but not necessarily speed. Physicians' perception of AI improved after implementation, highlighting its role as a "second reader" to support radiologists rather than replace them [7].

Sorin et al. conducted a systematic review that highlighted GAN application in various imaging modalities, including computed tomography, magnetic resonance imaging, and positron emission tomography. Physicians reported improvements in image quality, especially regarding reconstruction and noise removal, which facilitated more accurate diagnoses [8].

Wicaksono et al. evaluated GAN application in magnetic resonance angiography of the brain, demonstrating that GANs significantly improved image resolution and vessel visibility without compromising diagnostic sensitivity and specificity [9].

In ophthalmology, the study by Gunasekeran et al. revealed that most ophthalmologists are willing to use AI as an assistive tool, especially for conditions such as diabetic retinopathy, glaucoma, and AMD. Major perceived benefits include improved access to care and diagnostic accuracy, although concerns about medical liability and errors are still significant obstacles [10]. Another study by Kawai et al. demonstrated that AI can improve the quality of OCT-angiography images in patients with diabetic retinopathy by reducing background noise and increasing the clarity of blood vessels. This facilitates a more accurate and faster quantitative assessment, which has been welcomed by clinicians [11]. Mehdizadeh et al. investigated the use of GANs for noise removal in OCT images and found that combining GANs with texture loss improved image quality and was received by clinical ophthalmologists, who reported better visualization of pathological features in the images [12].

Despite the encouraging results, the study has some limitations that should be kept in mind. The size of the sample, with 147 physicians, may not comprehensively represent the opinion of the ophthalmology community, limiting the generalizability of the findings. The questionnaire used is original and has not been previously validated. Future studies should employ validated assessment instruments to objectively evaluate the acceptability of AI algorithms. Additionally, they should investigate the perceived acceptability of AI models applied to imaging-based biomarker identification and automated diagnostic report generation.

In addition, the subjectivity of the assessments, resulting from the qualitative nature of the perceptions of the clarity and usefulness of the AI-treated images, may have introduced bias into the responses. Variations in opinions may be related to differences in physician training and experience, previous exposure to the technology, and the interpretation and application of AI in different clinical contexts. Future research could explore these additional dimensions and evaluate longitudinally how the ongoing integration of AI in ophthalmology influences clinical perceptions and practices over time.

The practical implications of the results suggest that, although AI can potentially enhance the quality of OCT images, its adoption in clinical practice should be approached cautiously and contextually. Improved image clarity may represent a significant advancement for more accurate diagnosis and identification of critical biomarkers [13]. However, the lack of consensus on the usefulness of AI for clinical management highlights the need for a balanced approach that considers both technological advances and the clinical expertise of ophthalmologists. The successful integration of AI into clinical practice requires adequate training and the consideration that, although AI can improve image visualization, the final interpretation still depends significantly on clinical judgment [14-16].

## Conclusions

The findings of this study suggest implications for ophthalmic clinical practice based on the evaluators' reported perceptions. According to the participants, the use of AI algorithms for processing OCT images improved the visual clarity of retinal layers by reducing noise and enhancing structural sharpness, factors they considered important for supporting more accurate diagnoses. Nevertheless, the prevailing perception that AI does not substantially contribute clinically relevant information indicates that the adoption of this technology should be considered with caution, as these insights reflect subjective impressions rather than evidence of direct clinical impact.

Future research should aim to expand the sample size and include a detailed collection of demographic data from participants to enable a more in-depth analysis of doctors' opinions. Further longitudinal studies are recommended to assess the efficacy of the filter presented in this article for ophthalmologists and other AI tools that use images to produce reports.

Thus, AI image enhancement algorithms facilitate the identification of retinal structures. Furthermore, investigating the cost-benefit ratio of implementing AI in routine clinical practice is essential to assess the investment needed in technology and training about the perceived clinical benefits. Finally, conducting comparative studies to evaluate various AI algorithms and their respective effectiveness in improving the quality of OCT images will be instrumental in identifying the most effective tools for clinical practice and exploring the impact of AI on the identification of specific biomarkers.
